# Supplementary material for: The Systems Biology Research Tool: evolvable open-source software
Source: BMC Syst Biol. 2008 Jun 29;2:55. doi: 10.1186/1752-0509-2-55 (PMC2446383; doi:10.1186/1752-0509-2-55)
Supplement: Additional file 1 — SBRT Archive. An archive of the current version of the Systems Biology Research Tool. [file 1752-0509-2-55-S1.zip › sbrt-1.4.0/doc/developers_guide/api/sbrt/shell/io/class-use/TextOutputFile.html]

Uses of Class sbrt.shell.io.TextOutputFile


|  |  |  |
| --- | --- | --- |
| **Overview** \| **Package** \| **Class** \| **Use** \| **Tree** \| **Deprecated** \| **Index** \| **Help** | |  |
| PREV   NEXT | **FRAMES**    **NO FRAMES**     **All Classes** |  |


---


## **Uses of Class sbrt.shell.io.TextOutputFile**

| Packages that use TextOutputFile | |
| --- | --- |
| **sbrt.shell.io** | Provides classes and interfaces for reading and writing files. |
| **sbrt.shell.mng.fba** | Provides classes and interfaces for creating Flux Balance Analysis process managers. |
| **sbrt.shell.mng.graph\_theory** | Provides classes and for creating graph theoretical process managers. |

Uses of TextOutputFile in sbrt.shell.io

| Methods in sbrt.shell.io that return TextOutputFile | |
| --- | --- |
| `static TextOutputFile<java.lang.String>` | `TextOutputFile.getBasic(java.lang.String fileName)`             Returns a basic text output file. |
| `static TextOutputFile<java.lang.String>` | `TextOutputFile.getBasic(java.lang.String fileName, FileFormat fileFormat)`             Returns a basic text output file with the provided file format. |

Uses of TextOutputFile in sbrt.shell.mng.fba

| Methods in sbrt.shell.mng.fba that return TextOutputFile | |
| --- | --- |
| `static TextOutputFile<LinearComb<java.lang.String>>` | `RandomObjectiveManager.getOutputFile(ManagerInput<java.lang.String,java.lang.String> input, java.io.PrintWriter out, IrrevRxnFluxome fluxome)`             Returns an empty output file that is suitable for storing randomly generated objective functions. |
| `static TextOutputFile<LinearComb<java.lang.String>>` | `RandomObjectiveManager.getOutputFile(java.lang.String fileName, FileFormat format, java.io.PrintWriter out, IrrevRxnFluxome fluxome)`             Returns an empty output file that is suitable for storing randomly generated objective functions. |

Uses of TextOutputFile in sbrt.shell.mng.graph\_theory

| Methods in sbrt.shell.mng.graph\_theory that return TextOutputFile | |
| --- | --- |
| `static TextOutputFile<java.util.List<java.lang.String>>` | `CycleIdManager.getOutputFile(ManagerInput<java.lang.String,java.lang.String> input)`             Creates a returns an output file if one is specified in the provided input. |
| `static TextOutputFile<java.util.List<java.lang.String>>` | `CycleIdManager.getOutputFile(ManagerInput<java.lang.String,java.lang.String> input, java.io.PrintWriter out)`             Creates a returns an output file if one is specified in the provided input. |

---


|  |  |  |
| --- | --- | --- |
| **Overview** \| **Package** \| **Class** \| **Use** \| **Tree** \| **Deprecated** \| **Index** \| **Help** | |  |
| PREV   NEXT | **FRAMES**    **NO FRAMES**     **All Classes** |  |


---
